# Coral Gardens Reef, Belize: A refugium in the face of Caribbean-wide *Acropora* spp. coral decline

**DOI:** 10.1371/journal.pone.0239267

**Published:** 2020-09-30

**Authors:** Lisa Greer, Tara Clark, Tanner Waggoner, James Busch, Thomas P. Guilderson, Karl Wirth, Jian-xin Zhao, H. Allen Curran

**Affiliations:** 1 Geology Department, Washington and Lee University, Lexington, VA, United States of America; 2 School of Earth Atmospheric and Life Sciences, University of Wollongong, Wollongong, NSW, Australia; 3 Radiogenic Isotope Facility, School of Earth and Environmental Sciences, The University of Queensland, Brisbane, QLD, Australia; 4 Department of Earth Sciences, Dartmouth College, Hanover, NH, United States of America; 5 Center for Accelerator Mass Spectrometry, Lawrence Livermore National Laboratory, Livermore, CA, United States of America; 6 Ocean Sciences Department, University of California, Santa Cruz, CA, United States of America; 7 Geology Department, Macalester College, St. Paul, MN, United States of America; 8 Geosciences Department, Smith College, Northampton, MA, United States of America; Newcastle University, UNITED KINGDOM

## Abstract

Caribbean *Acropora* spp. corals have undergone a decline in cover since the second half of the twentieth century. Loss of these architecturally complex and fast-growing corals has resulted in significant, cascading changes to the character, diversity, and available eco-spaces of Caribbean reefs. Few thriving *Acropora* spp. populations exist today in the Caribbean and western North Atlantic seas, and our limited ability to access data from reefs assessed via long-term monitoring efforts means that reef scientists are challenged to determine resilience and longevity of existing *Acropora* spp. reefs. Here we used multiple dating methods to measure reef longevity and determine whether Coral Gardens Reef, Belize, is a refuge for *Acropora cervicornis* against the backdrop of wider Caribbean decline. We used a new genetic-aging technique to identify sample sites, and radiocarbon and high-precision uranium-thorium (U-Th) dating techniques to test whether one of the largest populations of extant *A*. *cervicornis* in the western Caribbean is newly established after the 1980s, or represents a longer-lived, stable population. We did so with respect for ethical sampling of a threatened species. Our data show corals ranging in age from 1910 (^14^C) or 1915 (^230^Th) to at least November 2019. While we cannot exclude the possibility of short gaps in the residence of *A*. *cervicornis* earlier in the record, the data show consistent and sustained living coral throughout the 1980s and up to at least 2019. We suggest that Coral Gardens has served as a refuge for *A*. *cervicornis* and that identifying other, similar sites may be critical to efforts to grow, preserve, conserve, and seed besieged Caribbean reefs.

## Introduction and background

Once prolific, *Acropora* coral species (*A*. *cervicornis* and *A*. *palmata*) are now increasingly rare and threatened across the tropical Western North Atlantic/Caribbean region. These fast-growing, branching scleractinian corals have been key in sustaining diverse ecological habitats in shallow marine environments and have long provided the architectural framework for expansive coral reef structures. Robust *Acropora*-dominant reefs were consistently present throughout the Pleistocene and Holocene epochs in the Caribbean province [1–5]. However, since the 1950s and 1960s, the structurally and functionally important *A*. *palmata* and *A*. *cervicornis* have suffered massive mortality and population declines of up to 98% across this region [3, 4, 6–11], although some studies suggest mortality may even predate this time [11–13]. Declines have been linked to multiple and sometimes synergistic drivers that include: temperature stress, overfishing, eutrophication from terrestrial sources, hurricanes, significant loss of sea urchins, and specifically for *Acropora* spp., white band disease, a coral disease that causes tissue death [6, 14–19]. Widespread recognition of their demise led *A*. *palmata* and *A*. *cervicornis* to be the first corals listed as threatened on the USA Endangered Species list [9]. Unabated loss of the fast-growing and architecturally complex *Acropora* spp. will have major impacts on structural integrity, rugosity, biodiversity, and live coral cover on future reefs in the face of climate and environmental change [13, 20–25]. How to preserve or promote *Acropora* spp. growth is an active topic of concern and identifying ‘successful’ or long-lived reefs may be essential to efforts to preserve or grow these corals in environments increasingly hostile to their success.

Much of the post-1980s coral literature initially focused on documenting reef decline, with many studies addressing global coral mortality, loss at key sites where acroporids once dominated, and/or the particular reasons for coral demise [6, 21, 26–35]. However, an increasingly robust literature on nurseries, reintroduction, conservation, and even assisted evolution of corals is emerging [36–52]. As the field expands from understanding why corals die to how we can promote restoration of these important ecological niches in a changing world, studies increasingly look to successful reefs to understand robustness, resilience, and temporal persistence [10, 33, 49, 53–67]. If we can identify the ‘reefs that work’ in spite of recent anthropogenic and environmental change we may be able to better characterize their features and facilitate reef expansion, cultivate nurseries, re-seed reefs, and conserve a dwindling ecological resource [63, 68, 69]; providing hope for the future of Caribbean coral reefs [70] and possibly beyond.

Identifying resilient reefs is not trivial. There are arguably only a few examples of extant ‘successful’ *A*. *cervicornis* reefs in the Caribbean region at present [40, 61, 71–75] and many of the reefs that have been subject to long-term monitoring are now in decline [30, 76–80]. Most examples of extant ‘healthy’ reefs have only recently been identified or monitored and of these, few have been monitored using quantitatively robust methodologies. Even when we identify what seems to be a thriving reef, there are few ways to assess temporal persistence, which is key to assessing resilience. There is also the complication of ‘shifting baselines’ where optimal ‘health’ has been arbitrarily determined by convenience or happenstance [81, 82]. In short, we have limited observational data on truly resilient acroporid reefs in the Caribbean region.

For the acroporid reefs recognized as thriving today, we are challenged to assess their longevity. Has a given reef persisted despite environmental or climate change, or is it rather a ‘new recruit’ that has taken over a formerly occupied habitat after a die-off? Is it an ephemeral short-lived reef that may not be establishing the kind of sturdy and complex framework on which long-lived reefs are built? While new recruits and ephemeral reefs may hold keys to natural seeding of reefs downstream, they may also operate differently than the well-documented, long-lived, and architecturally robust reefs of the recent geologic past [2, 4, 83–86]. Assessing acroporid persistence in modern reefs is also challenging with respect to sampling methodology and ethical concerns for the health and preservation of extant reefs, especially with respect to endangered species. In the not-too-distant past, scientists have cored through living reefs to sample older, underlying corals for dating purposes. Present awareness of the growing threats to reefs now gives us pause in potentially compromising the structural integrity of living reefs with heavy hydraulic coring equipment and tripods. Alternate methods of plucking dead coral rubble from within living canopy and carefully digging pits in areas devoid of living coral should be employed when possible.

Another fundamental challenge to determining persistence in modern reef growth lies in the temporal resolution offered by available dating methods. Complications with defining a precise (<10 years) calibrated age using radiocarbon exist because of the preformed age of surface ocean waters, the reservoir effect [87], and post-bomb values, essentially post-1957, which can yield non-unique solutions on either side of the post-bomb surface ocean peak values [e.g. 88]. Historically, very young samples have been challenging to date with U-Th because of the extremely low ^230^Th that is produced as well as potential biases caused by detrital ^230^Th. However, traditional methods such as radiocarbon and U-Th dating can complement each other [89–92]. Conventional ^230^Th can typically yield ages with two-sigma age errors of around +/- 20 years for mid Holocene [93] and +/- 1,000 years for late Pleistocene corals [94] using U-Th methods. The inability to account for initial ^230^Th sources of U-Th dated material can still lead to inaccurate age estimates with these methods [95–97]. Fortunately, increasingly improved U-Th dating techniques offer vastly refined precision in age ranges and avoid the issues that changing atmospheric radiocarbon concentration pitfalls pose [e.g., 98]. Clark et al. [97, 99] developed a U-Th dating method for Great Barrier Reef corals that returns a precision of less than a year for modern corals using rigorous cleaning techniques and by constraining local initial ^230^Th/^232^Th values. One new method for assessing the ‘age’ of clonal corals is to quantify the amount of somatic mutations found in very small portions of living coral tissue sampled for genetic analysis [100, 101]. This method yields a range of possible ages, but using only the conservative minimum genetic age estimate can still help to identify reefs that have veteran (long-lived) populations versus new recruits.

This project aims to contribute to a growing body of research on thriving reefs and to document a refugium for Caribbean acroporid corals. Here we determined the temporal persistence of a living acroporid reef in Belize dominated by *Acropora cervicornis* to investigate whether the *Acropora* spp. corals at Coral Gardens Reef are ephemeral, in recovery, or remnant populations that survived the Caribbean-wide collapse of recent decades. We used genetic data from Irwin et al. [101] to identify areas of the reef that may have long-lived (veteran) *A*. *cervicornis* corals. The temporal persistence of this population was confirmed by dating dead skeletons found both *in situ* and amongst the coral death assemblage using radiocarbon and precise U-Th dating methods. We did so with respect for ethical concerns regarding sampling methods and impacts on living coral. Our data suggest that the Coral Gardens Reef has served as a long-term refugium for *Acropora* spp. corals.

### Study area

**Fig 1:**
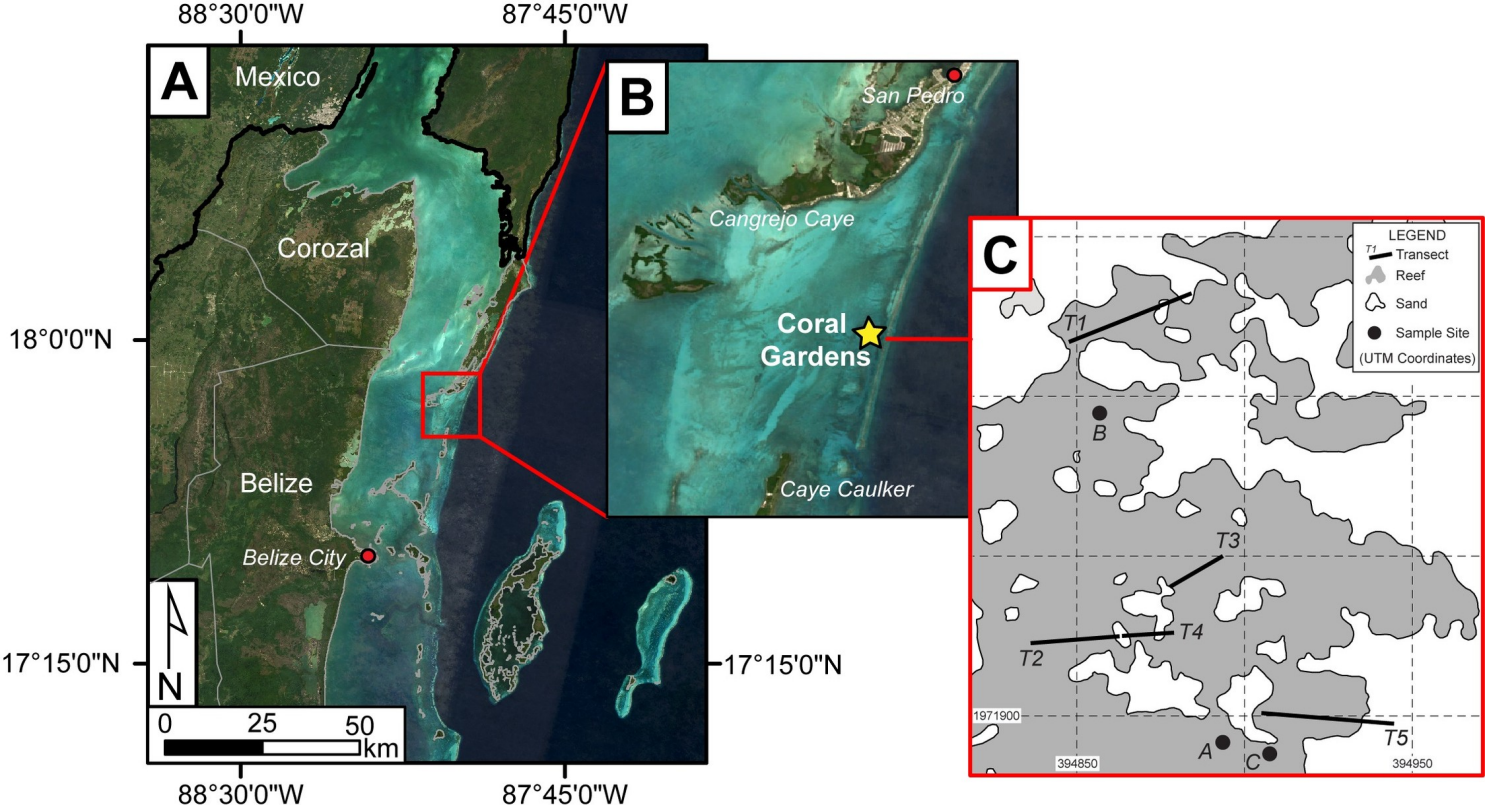
Location of sampling sites. A) Location map of the Coral Gardens Reef study area; B) Inset map of Coral Gardens located between Ambergris Caye (San Pedro) and Caye Caulker islands. C) Pits A, B, and C were excavated in areas of non-living reef framework. T1-T5 represent transect locations across patches of living *Acropora cervicornis*. Images used in A and B are modified from Landsat-8 imagery courtesy of the United States Geological Survey.

Coral Gardens Reef [17° 49’ 54.7644”, 87° 59’ 29.9743”], located approximately six kilometers southeast of the southern tip of Ambergris Caye, Belize, is situated inshore of the Mesoamerican Barrier Reef between Ambergris Caye and Caye Caulker (Fig 1A and 1B). Massive thickets of *A*. *cervicornis* cover approximately 7.5 hectares of this shallow back reef area, where water depth varies little and reaches a maximum depth of ~7 m. Coral Gardens contains one of the largest recorded extant acroporid populations in the Caribbean (Fig 1C; [61]). All three Caribbean *Acropora* species (*A*. *cervicornis*, *A*. *palmata*, and *A*. *prolifera*) are found here in patches interspersed with a variety of mixed massive and finger corals (primarily *Porites*, *Orbicella*, *Millepora*, and *Agaricia* spp.) and sandy areas. The outer perimeter of the area is commonly dominated by seagrass beds. The relatively monospecific thickets of branching corals (acroporids) vary in size but can reach diameters of up to 35 m.

This site has experienced the passage of over a dozen major tropical storms or hurricanes since c.1930. Recent major events include Category 4 Hurricane Keith in 2000 (landfall near Caye Caulker), Category 5 Hurricane Dean in 2007 (landfall just north of the Belize/Mexico border), Tropical Storm Karl in 2010 (landfall just north of Chetemal, Mexico), and Category 1 Hurricane Earl in 2016 (landfall on Ambergris Caye). Coral Gardens is not located proximal to any major rivers so flooding and/or sediment stress associated with these storms likely has not been a major issue. However, Coral Gardens is situated near a break in the reef crest, so it is potentially vulnerable to physical wave damage associated with high storm winds. Rainfall and sea surface temperature variability at this site is presumed similar to other sites in Belize where acroporids are rare. No mass-bleaching event has been observed on site from 2011 to summer 2019, although a mass bleaching event began in the fall of 2019. The effects of this event have not yet been evaluated.

## Materials and methods

### Site selection

**Fig 2:**
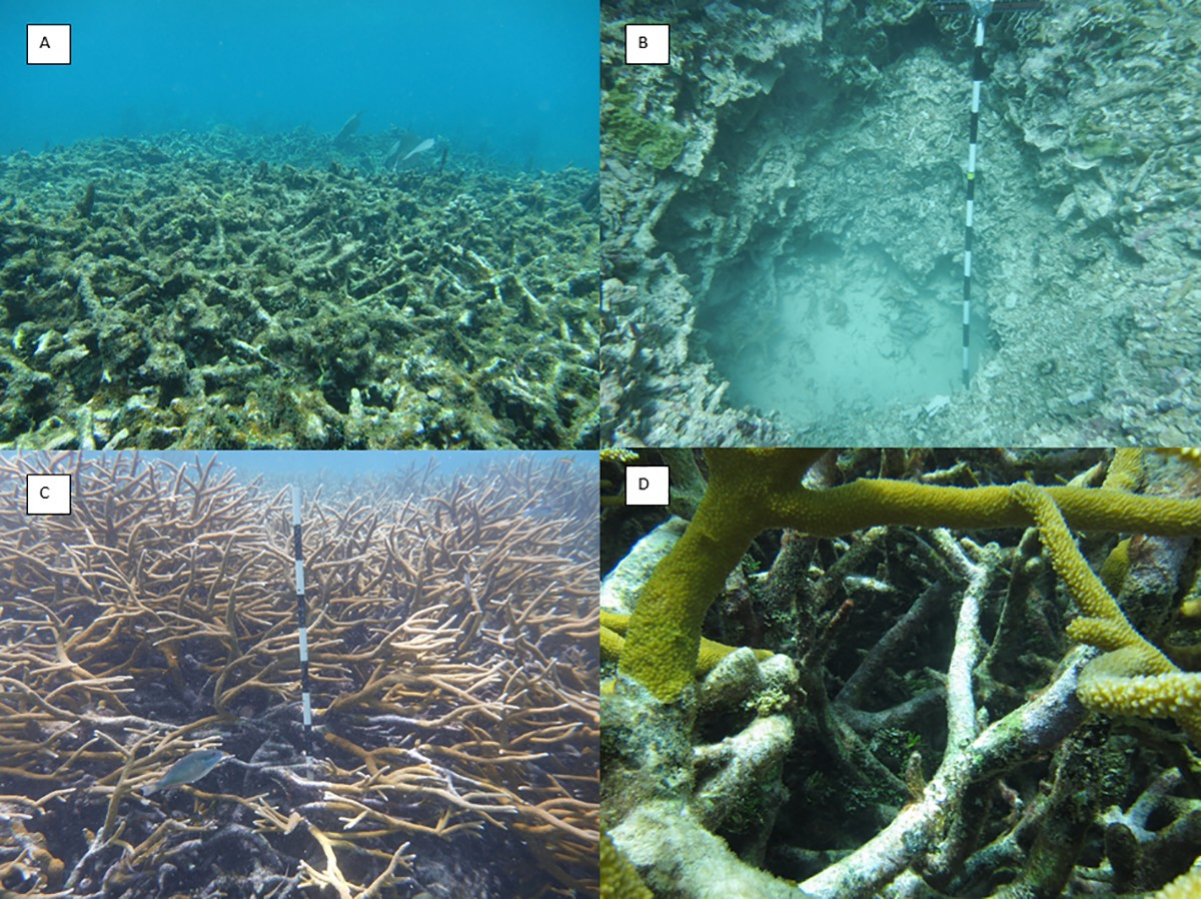
Sampling location and character. A) Non-living substrate at T5 prior to excavation (Photo: Lisa Greer); B) Excavation Pit C with vertical metric scale bar. Each black or white bar on the scale represents 10 cm; maximum depth of this pit was 2 m (Photo: Lisa Greer); C) Typical area for dead coral sampling and canopy structure at T5 (Photo: Lisa Greer); D) Open coral canopy facilitated non-destructive sampling of coral rubble within living coral patches (Photo: Lisa Greer).

In the summer of 2013 and 2014, small samples of living *Acropora cervicornis* coral from Coral Gardens were collected for determination of genetic composition. These data were used to age corals via a newly developed genetic analysis technique [100]. Results suggest Coral Gardens is composed of both veteran (long-lived) corals (clones spread via asexual fragmentation) and new recruits (reproduced via sexual reproduction; [101]). We used these data to establish target areas in the reef for sampling in 2015 and 2016 of pre-modern and recently dead coral skeletal material for radiometric dating. The three target areas were positioned within ~5 m of living reef framework (Fig 1C) and capped by *Acropora* coral that appeared to be ‘recently dead.’ The criteria for choosing these sampling locations were that they included no living *Acropora* spp. coral tissue but *in situ* growth positions of branches, encrustation by fleshy and calcareous algae, low taphonomic grade, and no other large coral species of over ~5 cm in diameter present in the immediate vicinity (Fig 2A).

### Fieldwork

In summer 2015 and 2016, divers carefully excavated three underwater pits measuring ~1 m in diameter using hammers, chisels, and buckets on SCUBA (Fig 2B). The sites were not suitable for traditional coring since some of the material was loosely consolidated, with sand or pore space in places that might cause collapse or stratigraphic mixing under high power hydraulic coring. This excavation method was also employed to minimize disruption to these shallow sites with any heavy or cumbersome equipment. In addition, our method allowed careful collection of more material for greater dating resolution. *In situ* samples totaling 191 fragments were collected in stratigraphic order in each pit at approximately 5 cm intervals (Table 1). However, the branching nature of *Acropora cervicornis* corals meant that the sampled coral fragments often had inclined orientations, so samples were not assumed to represent a perfectly linear timeline representing sequential growth. Pits A, B, and C were excavated to depths of ~1, ~1.2, and ~2 m, respectively, with each pit underlain by additional dead *A*. *cervicornis* rubble. All samples were cleaned of organic matter (mostly fleshy algae) using a chemical solution of 50% bleach by volume and dried.

**Table 1:** Number of samples collected, analyzed using XRD (X-ray Diffraction), SEM (Scanning Electron Microscopy), stable isotope, radiocarbon and U-Th analyses, and results of radiocarbon and U-Th dating. Twenty- six samples were cross-dated with radiocarbon and U-Th methods.

Field Site	Samples (n)	XRD (n)	SEM (n)	Stable Isotope (n)	^14^C Dated (n)	^14^C Age Max	^14^C Age Min	Avg ^14^C Error (2σ)	U-Th Dated (n)	U-Th Age Max	U-Th Age Min	Avg STD Error
**Pit**	191	35	10	24	39	1973	1910	45.1	10	1996.8	1915.3	1.6
**Canopy**	41	4	4	25	23	1973	1970	6	25	2015	1982.7	1.1

In 2016, 41 additional samples of dead *A*. *cervicornis* rubble were collected from underneath or within the living reef framework at site T5 using a 1.5 m extendable grip and grab (rubbish picker) tool (Table 1; Fig 2C), rather than coring through live reef. Samples were collected based on accessibility to avoid any harm to living coral. This was possible because of the relatively large and open *A*. *cervicornis* canopy (Fig 2D). One small sample (~2 cm in length) of living *A*. *cervicornis* was collected and used for U-Th age calibration purposes. All necessary permits (CITES and Belize Fisheries Management Unit) were obtained for the collection of all samples in this study, which complied with all relevant regulations. Permits obtained for field collection include Permit # 0000033–15 from the Belize Fisheries Department Ministry of Forestry, Fisheries & Sustainable Development (and renewals), CITES International Trade in Endangered Species of Wild Fauna and Flora Permit/Certificate No. 5673, and CITES International Trade in Endangered Species of Wild Fauna and Flora Post Entry number: 2016075 after compliance interview (Dec Control Num: 2016876077).

### Sample preparation

**Fig 3:**
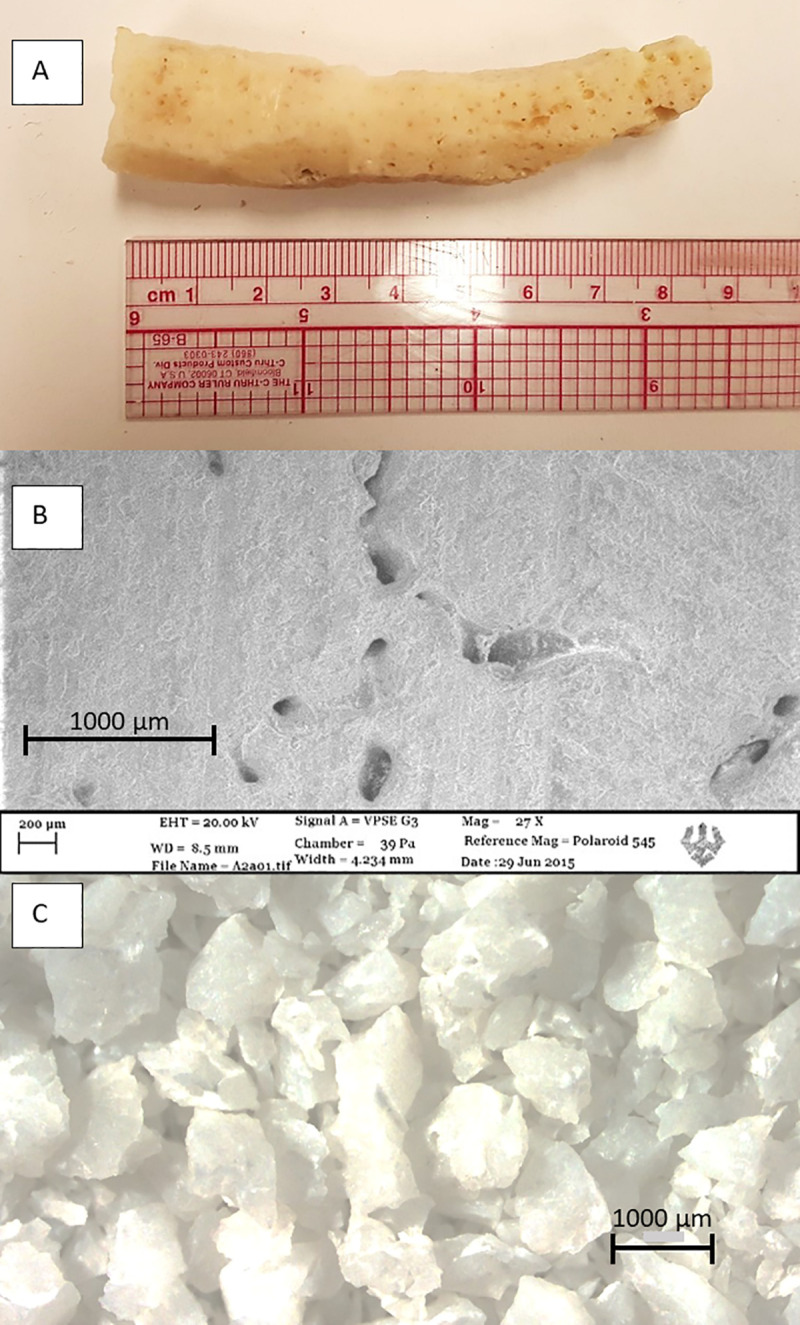
Sample quality. A) *Acropora cervicornis* rubble sample with outer surface removed prior to screening (Photo: Tanner Waggoner); B) Scanning electron microscope image of a coral surface with no visible signs of alteration (Photo: Tanner Waggoner); C) Crushed aragonite coral skeleton free from contaminants viewed from under a binocular microscope (Photo: Tanner Waggoner).

In the lab, the outer encrusted and bored surfaces of corals were removed using a lapidary saw to expose unaltered aragonite skeletal carbonate (Fig 3A). Sample quality was assessed for a subset of samples using a Diano 2100E X-ray diffractometer (XRD), a Zeiss EVO MA15 scanning electron microscope (SEM) with electron backscatter diffraction capabilities (EBSD), and an Olympus IX51/Nixon DS-U2 binocular microscope at Washington and Lee University (Table 1; Fig 3B and 3C). Only pure aragonite samples, free of any visible anomalies, impurities, borings, encrustations, or discoloration were selected for dating (Fig 3B and 3C).

### Radiocarbon dating

In preparation for radiocarbon analyses, 62 aragonite samples of approximately 1 cm^3^ volume were crushed using mortar and pestle and sieved to approximately <2 mm-sized fragments. Ten mg of the cleanest fragments from these larger samples were lightly leached in weak hydrochloric acid, rinsed, and dried prior to being placed in individual vacutainers and evacuated to <1x10^-3^ Torr at the Lawrence Livermore National Laboratory Center for Accelerator Mass Spectrometry. Calcium carbonate was hydrolyzed using 85% orthophosphoric acid at 90°C. Resultant CO_2_ was purified cryogenically and reduced to graphite at 570°C in the presence of an iron catalyst and a stoichiometric excess of hydrogen. Carbon-14 content was determined using accelerator mass spectrometry. Data were reported as fraction modern and conventional radiocarbon age (years before present (BP) defined as 1950 AD) according to the convention of Stuiver and Polach [102], including a background correction based on ^14^C-free calcite, and measured *δ*^13^C values for each sample. Data were calibrated to calendar years using Oxcal Version 4.2 against the Marine 13 calibration curve [103, 104]. A code for Oxcal v4.2 that accounts for the post-bomb local/regional offset in radiogenic carbon at Coral Gardens Reef [88] was created using data from nearby Glovers Reef (16° 50' N, 87° 50' W).

### U-Th dating

Thirty-five approximately 1 cm^3^ coral fragments were prepared for high-precision U-Th dating following rigorous cleaning procedures after Clark et al. [97] designed to eliminate detrital contaminants with high concentrations of ^232^Th within the aragonite skeletal material. Each sample was crushed using an agate mortar and pestle, and ~1 mm-sized fragments were soaked overnight in 10% H_2_O_2_ and Milli-Q water_._ Samples were then rinsed with Milli-Q water and centrifuged for 15 min at 4000 rpm in 10% H_2_O_2_ solution, and residual hydrogen peroxide solution was extracted. Remaining fragments were rinsed with Milli-Q water and sonicated multiple times until the solution was clear. Samples were then dried on a hotplate at 40°C overnight. Each sample was examined and photographed using the Olympus IX51/Nixon binocular microscope. From each sample, ~150 mg of clean, unaltered aragonite fragments was selected for U-Th dating.

Uranium and thorium were separated and purified using ion-exchange column chemistry procedures modified from Edwards et al. [105], and samples were dated at The University of Queensland Radiogenic Isotope Facility following methods described by Clark et al. [97, 99]. All Th-separate solutions and representative U-separate solutions were screened on a Thermo X-Series II Quadrupole ICP-MS to determine the concentration of U prior to measurement on a Nu Plasma Multi-Collector Inductively Coupled Plasma Mass Spectrometer (MC-ICP-MS). The pre-screening allows mixing of the entire Th-separate solution with a portion of the U-separate solution to achieve the optimal signal intensities for high-precision MC-ICP-MS measurements of U and Th isotope ratios at high throughput. The MC-ICP-MS instrument has deceleration lenses behind each of the two active secondary electron multipliers (named IC0 and IC2) to substantially increase abundance sensitivity to allow measurement of these young (<100 yrs old) samples. U and Th isotopes were measured in two sequences of one mass difference to allow ^230^Th and ^229^Th determined on IC2 and ^234^U and ^233^U on IC0, respectively, as described in detail by Clark et al. [97, 99]. The U-Th age data were corrected for two isotopically distinct sources of non-radiogenic ^230^Th; soluble (only absorbed into the coral skeleton during growth) and insoluble (incorporated into the skeletal matrix of the coral either during the growth or post-mortem), using a two-component mixing model, also described by Clark et al. [97]. Site specific ^230^Th_0_ values for the hydrogenous and detrital components obtained from Coral Gardens Reef are listed in S2 Table. The suitability of these values was confirmed by U-Th dating one modern coral of known age (collected in 2015). Twenty-seven coral samples were cross-dated using both radiocarbon and U-Th methods.

## Results

A total of 191 non-living *Acropora cervicornis* samples were collected from pit excavations, with 110 of these samples collected from Pit C. Forty-one additional non-living samples were retrieved from within or beneath the reef framework at T5, as well as one small (~2 cm length) living sample for calibration purposes, giving a total of 232 samples from Coral Gardens. X-ray diffraction and visual inspection of a representative sampling of 35 excavated and four samples retrieved from beneath the living canopy revealed pure aragonite composition, with no signs of diagenetic alteration or contamination of any sample by non-aragonite material. Ten excavated and four samples retrieved from beneath the living coral canopy were randomly chosen as representative of the sample population and were screened for diagenetic fingerprints (e.g. rhombic crystal shape, dissolution pitting, or presence of non-aragonite material contaminants) using SEM/EBSD to further confirm the purity of these samples and suitability for radiometric dating. No secondary aragonite was observed in samples.

**Fig 4:**
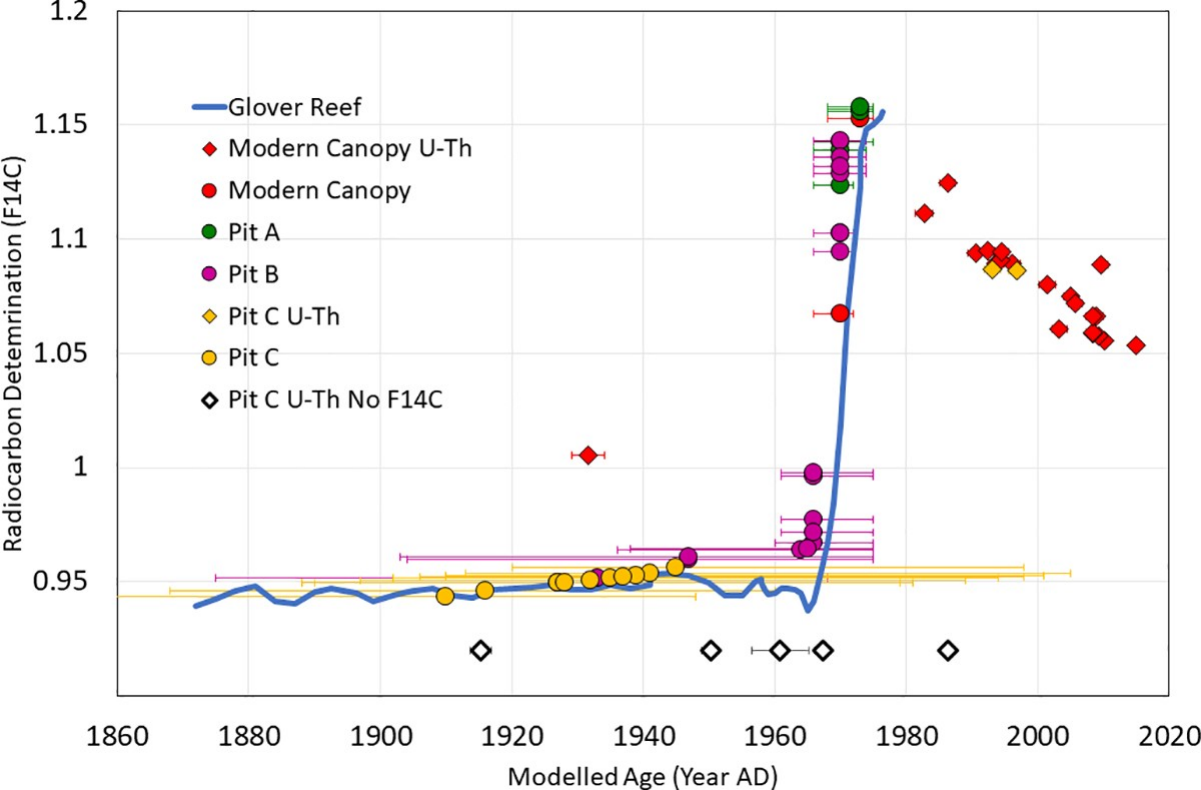
Radiocarbon and U-Th data. All ^230^Th and radiocarbon dates from Pits A, B, C and the modern canopy at Coral Gardens, color-coded by sample population and with standard error plotted on the radiocarbon calibration curve from Glovers Reef, Belize [88]. Circles represent radiocarbon dates and diamonds represent ^230^Th ages. ^230^Th ages are used in place of all radiocarbon ages for cross-dated samples. Unfilled diamonds represent samples with ^230^Th ages that were not radiocarbon dated and therefore do not have a F14C value. The ages of these samples are indicated below the radiocarbon calibration curve for Glovers Reef [88] for ease of visualization.

Calibrated radiocarbon ages of 62 coral samples range between 1910 to 1973 AD with 2σ age error ranges between 6 and 45 years (Table 1). U-Th ages of 34 samples range between 1915.3 ±1.6 to 1996.8 ±1.2 AD for excavated samples and 1982.7 ±1.3 to 2011.4 ±0.9 AD for samples retrieved from beneath the living canopy, as well as a living coral from 2015 (Table 1, Fig 4). Additional information on radiocarbon and U-Th results can be found in S1 and S2 Tables. The δ ^238^U and U(ppm) values of all samples are similar to that of modern seawater and modern coral samples [13, 99], respectively, which also suggests little to no alteration. One additional sample from within the coral canopy dated to 1931.6 ±2.6 AD (sample F6a_BZ-CG-TW15). While we did not exclude this data point, we consider the possibility of contamination or reworking for this sample, noting the low Δ^14^C value for modern canopy corals (S1 Table) and the slightly high ^232^Th value compared to the other samples (S2 Table). A comparison of twenty-seven samples analyzed by both U-Th and ^14^C methods plotted against a radiocarbon calibration from Belize [88] enabled an extension of the radiocarbon calibration curve for this area (Fig 4). U-Th dating of one modern coral of known age (collected in 2015) revealed an age of 2015.1 ±0.9 AD following correction for initial ^230^Th/^232^Th using the two-component correction scheme applied to Great Barrier Reef coral samples [97].

## Discussion

**Fig 5:**
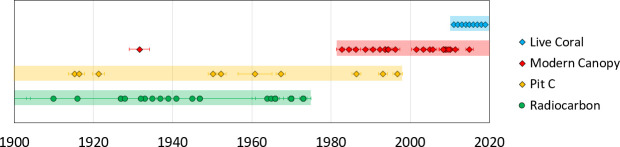
Continuity of *Acropora cervicornis* growth at Coral Gardens. All U-Th and radiocarbon ages of samples from Pit C and from within the modern living canopy. Note the substantial chronological overlap between corals from within living coral canopy and beneath the death assemblage (boxed) during the time of Caribbean-wide demise of acroporids. Abundant living corals were observed at least annually from 2011 to 2019.

**Fig 6:**
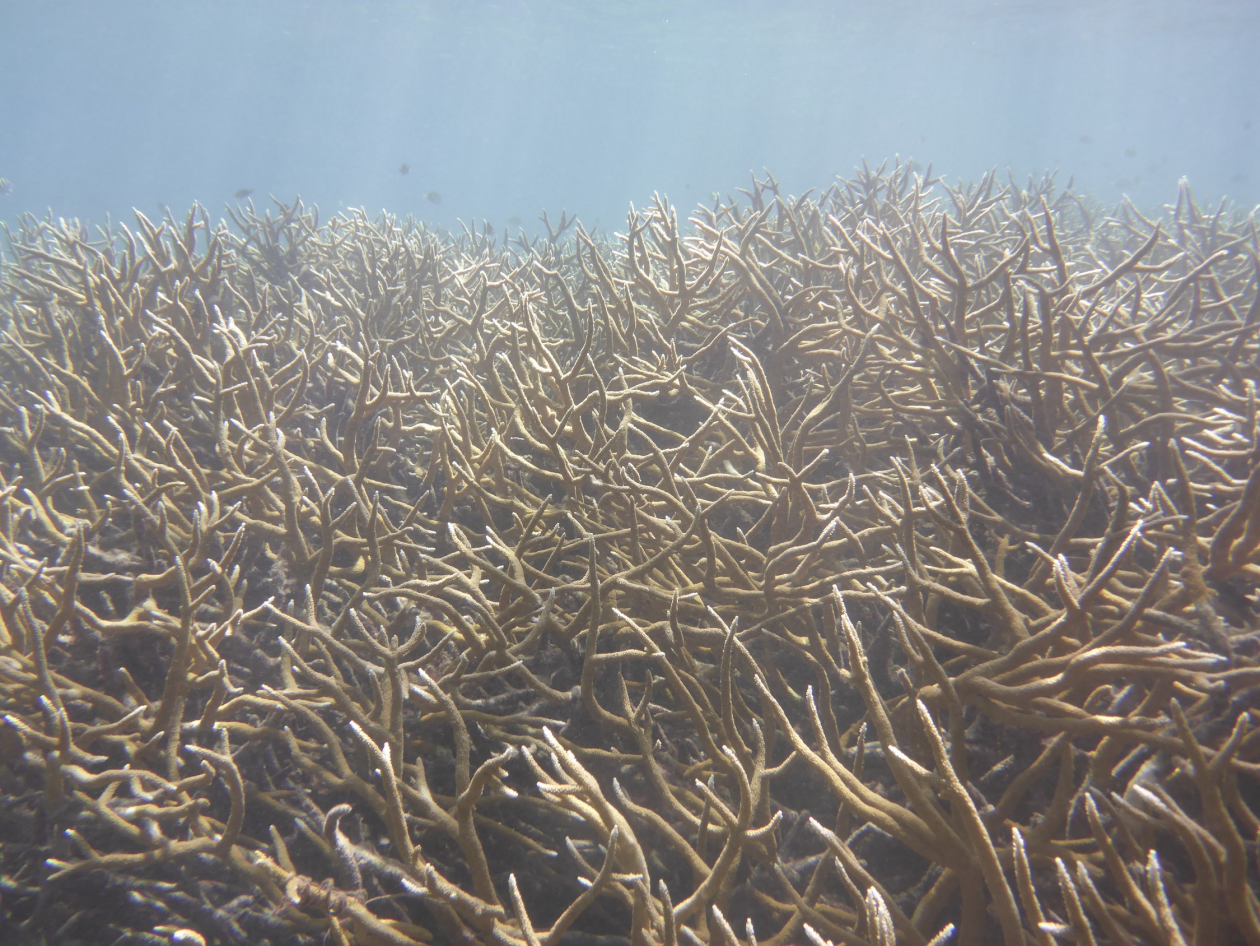
Living coral canopy. Typical view of flourishing live coral at location T5 in November 2019 (Photo: Lisa Greer).

A crucial step in transitioning from documenting coral decline to identifying areas of reef resilience is determining temporal persistence of reefs with robust resolution. Our data suggest Coral Gardens has served as a refugium for *Acropora* spp. corals during recent widespread decline of Caribbean reefs. *A*. *cervicornis* corals have persisted and grown for more than 100 years at the sampling locations of Pit C and T5 at Coral Gardens Reef from 1910 ^14^C or 1915 ^230^Th to 2015 (Figs 4 and 5), and were still thriving as of November 2019 (Fig 6). Since additional *A*. *cervicornis* rubble existed at the bottom of each pit (we were limited in time and scope to sample further), it is possible and seems highly likely that these corals were established for a significantly longer period of time. While we cannot guarantee that there were no temporary die-offs earlier in the record (with less age control and fewer dated samples deeper in the excavation pits), we have no geological evidence to support a hiatus in reef growth. The branching morphology of the corals also does not permit a strict linear interpretation, however, there were no apparent breaks or any observable hiatus in the pit stratigraphy. Additionally, no distinct layers of sediment, hardgrounds, high taphonomic-grade corals, or calcareous cement layers were encountered that would suggest discontinuous growth of corals at Coral Gardens. The radiometric ages obtained from the excavated corals are in stratigraphic order and overlap with the ages of the corals retrieved from within the living coral canopy, suggesting continuous *A*. *cervicornis* growth before, during, and after formation of the death assemblage capping Pit C. Corals of similar age are also found between the interstices of the modern living reef and beneath the mid-1990s death assemblage. The continuity of coral growth in the 1950s through 1980s suggests that no coral die-off event occurred at Coral Gardens in contrast to reefs elsewhere, with ages obtained from the death assemblage (such as in Pit C) postdating the onset of mortality at most other Caribbean locations (~1980s or earlier). We also see no evidence of a peak in mortality associated with the 1998 El Niño year that resulted in bleaching events elsewhere in Belize [106]. This does not mean that an El Niño, hurricane, and/or other stress events did not result in coral death, only that there is no evidence of a massive die-off or collapse in the record retrieved from Coral Gardens Reef.

Many definitions of the term refugia refer to persistence of a species for millennia, and through major climate changes [107]. The best measure of longevity in reefs comes from the fossil record. Many studies have shown that acroporids can be long established and resilient in the face of natural climate and environmental change. Age data from well-exposed reef crests provide a wealth of information about the general persistence of *Acropora cervicornis* through the Holocene and Pleistocene [1, 2, 4, 13, 84, 108]. At numerous sites in the Caribbean, including Florida [109], Mexico [84], the Bahamas [110], Barbados [4, 83], Jamaica [3, 111], the Gulf of Mexico [112, 113], US Virgin Islands [86, 114], and the Dominican Republic [5], fossilized reefs from the earlier Holocene offer impressive examples of the reef-building capacity of *A*. *cervicornis* [1, 83, 84, 108, 115].

Pre-modern acroporid corals have been well documented in Belize. Using core data from shallow-water reefs in southern Belize, Aronson and Precht [6] suggested that prior to the 1980s *A*. *cervicornis* was a primary architectural component of Belizean reefs for the past three millennia. While most of these studies can address the overall timespan of reef occupation at a given site, the paucity of undisturbed core sequences including datable reef materials does prevent high-resolution temporal analysis and the ability to answer more complex ecological questions such as the timing of ecological shifts, disturbance and recovery, and the ability to link them with associated drivers [116–118]. The lack of easily accessible continuous sequences of fossil corals, field and analytical costs, and sample quality often prohibit such analyses.

The question of why Coral Gardens Reef is a refugium is not addressed in this study. Detailed annual monitoring of Coral Gardens from 2011–2019 has yet to precisely reveal why this site has escaped the devastating demise that acroporids at so many other sites in the Caribbean have experienced. Coral Gardens has been subject to the impacts of tropical storm and hurricane force wind and wave action. It has experienced the escalating temperatures and El Niño events seen globally in recent years. The genetic diversity of *Acropora* spp. corals at this site is not unusually high [101].

**Fig 7:**
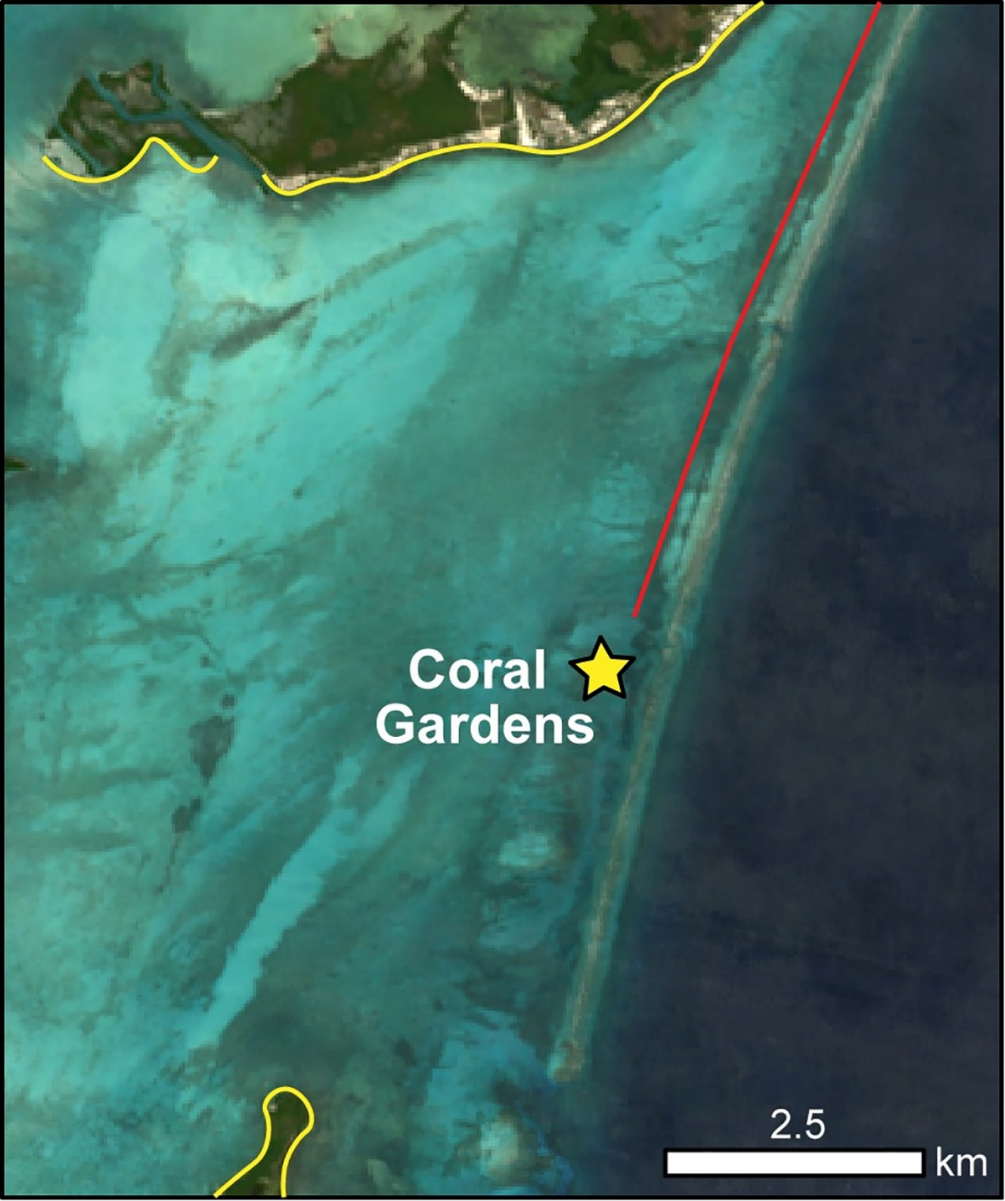
Coral Gardens in relation to reef crest. Red indicates the line of shallow reef crest off Ambergris Caye. Yellow indicates outline of Ambergris Caye (north) and Caye Caulker (south). Landsat-8 imagery is courtesy of the United States Geological Survey.

However, Coral Gardens did not experience any significant bleaching events from 2011 to June 2019, the site sees very little recreational traffic (situated between heavily visited marine protected areas), and the abundant and very shallow acroporid thickets discourage heavy fishing pressure. While *Diadema* urchins are still rare, overall urchin abundance (primarily *Echinometra viridis*) is high at this site and it seems that herbivores are keeping algae in check at Coral Gardens [119]. It is possible that location next to a break in the reef crest (potentially flushing excess or delivering valuable nutrients) (Fig 7) and distance from the impacts of riverine terrestrial influx may contribute to the good health of Coral Gardens. Regardless, the temporal persistence of endangered acroporid corals at Coral Gardens from at least 1915 to 2019 AD suggests that this site is an area that warrants sound and thoughtful conservation choices. As reef restoration science moves beyond studying the demise of *Acropora* spp. corals toward ever more serious restoration and conservation efforts, Coral Gardens may be an important site to consider for serious efforts aimed at preservation of this critical acroporid coral habitat.

## Conclusions

Our data show a temporal persistence in *Acropora cervicornis* growth at Coral Gardens Reef for over 100 years. Regardless of whether or not there were small gaps in the growth record in the early-mid 1900’s, *A*. *cervicornis* persisted at this site through the devastating die-off that plagued most of the Caribbean from the 1960s through the 1980s. This suggests that Coral Gardens served as a refugium for *A*. *cervicornis*, potentially one of very few areas where this species has escaped collapse. This study is one of a very few to date which examines the persistence of modern acroporid corals at fine-scale resolution, and it demonstrates how this approach can be valuable in assessing where corals have survived naturally–which should be useful to conservation, preservation, and nursery initiatives globally.

Hope remains that enough natural coral refugia exist to withstand collapse long enough for local, regional, or global intervention and/or stabilization to occur [45, 63, 67, 69, 70]. Coral Gardens Reef, Belize, is one candidate for persistence of endangered *Acropora* spp. corals, but for how long, we do not know. It is critical to understand natural working systems if we hope to promote persistence in coral communities, recruits for transplantation, best practices in management, and an understanding of human/environment interactions as the oceans venture into a climate-stressed future.

## Supporting information

S1 Table Radiocarbon analytical data. (DOCX)

S2 Table U-Th analytical data. (DOCX)
