# Supplementary material for: Coral Gardens Reef, Belize: A refugium in the face of Caribbean-wide Acropora spp. coral decline
Source: PLoS One. 2020 Sep 30;15(9):e0239267. doi: 10.1371/journal.pone.0239267 (PMC7526931; doi:10.1371/journal.pone.0239267)
Supplement: S1 Table — (DOCX) [file pone.0239267.s001.docx]

Table S1. Radiocarbon age data for 62 dead coral samples collected from Coral Gardens, Belize.

| Sample # | CAMS ID | Sample Name | Sample Location | Fraction Modern | ± | 14C age | ± | Lower Age (AD) | Upper Age (AD) | Modelled Age (Median) | Acomb | Age Range |
| --- | --- | --- | --- | --- | --- | --- | --- | --- | --- | --- | --- | --- |
| 1 | 173631 | F10a-BZ-CG-TW15 | Modern Canopy | 1.0556 | 0.0032 | >Modern |  | 1966 | 1972 | 1970 | 85.6 | 6 |
| 2 | 173627 | F1a-BZ-CG-TW15 | Modern Canopy | 1.0892 | 0.0028 | >Modern |  | 1966 | 1972 | 1970 | 85.6 | 6 |
| 3 | 173637 | F1b-BZ-CG-TW15 | Modern Canopy | 1.0891 | 0.0027 | >Modern |  | 1966 | 1972 | 1970 | 85.6 | 6 |
| 4 | 173634 | F3a-BZ-CG-TW15 | Modern Canopy | 1.0663 | 0.0028 | >Modern |  | 1966 | 1972 | 1970 | 85.6 | 6 |
| 5 | 173581 | F6a-BZ-CG-TW15 | Modern Canopy | 1.0052 | 0.0026 | >Modern |  | 1966 | 1972 | 1970 | 85.6 | 6 |
| 6 | 171043 | F7a-BZ-CG-TW15 | Modern Canopy | 1.0536 | 0.0035 | >Modern |  | 1966 | 1972 | 1970 | 85.6 | 6 |
| 7 | 173579 | F8a-BZ-CG-TW15 | Modern Canopy | 1.0576 | 0.0026 | >Modern |  | 1966 | 1972 | 1970 | 85.6 | 6 |
| 8 | 173583 | F9a-BZ-CG-TW15 | Modern Canopy | 1.0751 | 0.0029 | >Modern |  | 1966 | 1972 | 1970 | 85.6 | 6 |
| 9 | 173584 | T5Eb-BZ-CG-TW15 | Modern Canopy | 1.0719 | 0.0037 | >Modern |  | 1966 | 1972 | 1970 | 85.6 | 6 |
| 10 | 173580 | T5Ed-BZ-CG-TW15 | Modern Canopy | 1.0589 | 0.0028 | >Modern |  | 1966 | 1972 | 1970 | 85.6 | 6 |
| 11 | 173585 | T5Ee-BZ-CG-TW15 | Modern Canopy | 1.0594 | 0.0026 | >Modern |  | 1966 | 1972 | 1970 | 85.6 | 6 |
| 12 | 173628 | T5Ef-BZ-CG-TW15 | Modern Canopy | 1.0663 | 0.0026 | >Modern |  | 1966 | 1972 | 1970 | 85.6 | 6 |
| 13 | 173622 | T5Na-BZ-CG-TW15 | Modern Canopy | 1.0803 | 0.0026 | >Modern |  | 1966 | 1972 | 1970 | 85.6 | 6 |
| 14 | 173630 | T5Nc-BZ-CG-TW15 | Modern Canopy | 1.0887 | 0.0029 | >Modern |  | 1966 | 1972 | 1970 | 85.6 | 6 |
| 15 | 173623 | T5Sd-BZ-CG-TW15 | Modern Canopy | 1.1248 | 0.0030 | >Modern |  | 1966 | 1972 | 1970 | 85.6 | 6 |
| 16 | 173636 | T5Sf-BZ-CG-TW15 | Modern Canopy | 1.0937 | 0.0028 | >Modern |  | 1966 | 1972 | 1970 | 85.6 | 6 |
| 17 | 173582 | T5Si-BZ-CG-TW15 | Modern Canopy | 1.1112 | 0.0027 | >Modern |  | 1966 | 1972 | 1970 | 89.2 | 6 |
| 18 | 173632 | T5Wa-BZ-CG-TW15 | Modern Canopy | 1.0605 | 0.0026 | >Modern |  | 1966 | 1972 | 1970 | 85.6 | 6 |
| 19 | 173633 | T5Wb-BZ-CG-TW15 | Modern Canopy | 1.0950 | 0.0027 | >Modern |  | 1966 | 1972 | 1970 | 85.6 | 6 |
| 20 | 173629 | T5Wc-BZ-CG-TW15 | Modern Canopy | 1.0909 | 0.0041 | >Modern |  | 1966 | 1972 | 1970 | 85.6 | 6 |
| 21 | 173635 | T5We-BZ-CG-TW15 | Modern Canopy | 1.0943 | 0.0027 | >Modern |  | 1966 | 1972 | 1970 | 85.6 | 6 |
| 22 | 167990 | 35-BZ-CG-JB14 | Modern Canopy | 1.0674 | 0.0037 | >Modern |  | 1966 | 1972 | 1970 | 85.6 | 6 |
| 23 | 167991 | 43-BZ-CG-JB14 | Modern Canopy | 1.1527 | 0.0040 | >Modern |  | 1968 | 1975 | 1973 | 97.2 | 92 |
| 24 | 167098 | 2A-BZ-CG-JB14 | Pit A | 1.1234 | 0.0039 | >Modern |  | 1966 | 1972 | 1970 | 97.7 | 92 |
| 25 | 167984 | 35A-BZ-CG-JB14 | Pit A | 1.1571 | 0.0047 | >Modern |  | 1968 | 1975 | 1973 | 95 | 92 |
| 26 | 167985 | 36A-BZ-CG-JB14 | Pit A | 1.1559 | 0.0041 | >Modern |  | 1968 | 1975 | 1973 | 87.6 | 91 |
| 27 | 167986 | 39A-BZ-CG-JB14 | Pit A | 1.1427 | 0.0046 | >Modern |  | 1966 | 1975 | 1970 | 92.1 | 93 |
| 28 | 167987 | 40A-BZ-CG-JB14 | Pit A | 1.1388 | 0.0043 | >Modern |  | 1966 | 1974 | 1970 | 95.2 | 92 |
| 29 | 167096 | 41A-BZ-CG-JB14 | Pit A | 1.1578 | 0.0040 | >Modern |  | 1968 | 1975 | 1973 | 79.6 | 7 |
| 30 | 167097 | 2B-BZ-CG-JB14 | Pit B | 1.1288 | 0.0042 | >Modern |  | 1966 | 1974 | 1970 | 74.6 | 8 |
| 31 | 167980 | 3B-BZ-CG-JB14 | Pit B | 1.1430 | 0.0040 | >Modern |  | 1966 | 1974 | 1970 | 74.6 | 8 |
| 32 | 167981 | 6B-BZ-CG-JB14 | Pit B | 1.1358 | 0.0040 | >Modern |  | 1966 | 1974 | 1970 | 85.6 | 6 |
| 33 | 167982 | 8B-BZ-CG-JB14 | Pit B | 1.1319 | 0.0040 | >Modern |  | 1966 | 1974 | 1970 | 78.4 | 14 |
| 34 | 167983 | 14B-BZ-CG-JB14 | Pit B | 1.1026 | 0.0041 | >Modern |  | 1966 | 1972 | 1970 | 98 | 92 |
| 35 | 167995 | 17B-BZ-CG-JB14 | Pit B | 1.0944 | 0.0039 | >Modern |  | 1966 | 1972 | 1970 | 97.4 | 93 |
| 36 | 167996 | 18B-BZ-CG-JB14 | Pit B | 1.1026 | 0.0050 | >Modern |  | 1966 | 1972 | 1970 | 90.7 | 93 |
| 37 | 167997 | 22B-BZ-CG-JB14 | Pit B | 0.9773 | 0.0035 | 185 | 30 | 1961 | 1975 | 1966 | 95.5 | 92 |
| 38 | 167998 | 25B-BZ-CG-JB14 | Pit B | 0.9673 | 0.0034 | 265 | 30 | 1960 | 1975 | 1966 | 98.7 | 92 |
| 39 | 167999 | 29B-BZ-CG-JB14 | Pit B | 0.9718 | 0.0035 | 230 | 30 | 1961 | 1975 | 1966 | 96.9 | 92 |
| 40 | 168000 | 30B-BZ-CG-JB14 | Pit B | 0.9600 | 0.0034 | 330 | 30 | 1904 | 1975 | 1947 | 97.5 | 92 |
| 41 | 168001 | 34B-BZ-CG-JB14 | Pit B | 0.9608 | 0.0040 | 320 | 35 | 1903 | 1975 | 1947 | 97.9 | 92 |
| 42 | 168002 | 35B-BZ-CG-JB14 | Pit B | 0.9638 | 0.0034 | 295 | 30 | 1936 | 1975 | 1964 | 85.6 | 6 |
| 43 | 168003 | 37B-BZ-CG-JB14 | Pit B | 0.9646 | 0.0034 | 290 | 30 | 1938 | 1975 | 1965 | 79.6 | 7 |
| 44 | 168004 | 39B-BZ-CG-JB14 | Pit B | 0.9964 | 0.0036 | 30 | 30 | 1961 | 1975 | 1966 | 77.3 | 8 |
| 45 | 167339 | 39BQA-BZ-CG-JB14 | Pit B | 0.9977 | 0.0040 | 20 | 35 | 1961 | 1975 | 1966 | 74.6 | 8 |
| 46 | 167095 | 40B-BZ-CG-JB14 | Pit B | 0.9515 | 0.0033 | 400 | 30 | 1875 | 1968 | 1933 | 85.6 | 6 |
| 47 | 173624 | A1b-BZ-CG-TW15 | Pit C | 1.0862 | 0.0032 | >Modern |  | 1966 | 1972 | 1970 | 76.4 | 15 |
| 48 | 171090 | A2a-BZ -CG-TW15 | Pit C | 1.0868 | 0.0027 | >Modern |  | 1966 | 1972 | 1970 | 78 | 14 |
| 49 | 171045 | I3a-BZ-CG-TW15 | Pit C | 0.9509 | 0.0028 | 395 | 30 | 1876 | 1932 | 1932 | 81.9 | 71 |
| 50 | 171046 | I9a-BZ-CG-TW15 | Pit C | 0.9496 | 0.0028 | 405 | 30 | 1875 | 1927 | 1927 | 82.7 | 72 |
| 51 | 171047 | K7a-BZ-CG-TW15 | Pit C | 0.9515 | 0.0028 | 375 | 30 | 1877 | 1935 | 1935 | 76.1 | 39 |
| 52 | 171036 | L5c-BZ-CG-TW15 | Pit C | 0.9565 | 0.0028 | 350 | 30 | 1879 | 1945 | 1945 | 75.8 | 37 |
| 53 | 171042 | L6a-BZ-CG-TW15 | Pit C | 0.9536 | 0.0028 | 365 | 30 | 1877 | 1941 | 1941 | 78 | 14 |
| 54 | 171092 | M4a BZ -CG-TW15 | Pit C | 0.9462 | 0.0029 | 435 | 30 | 1872 | 1916 | 1916 | 78.1 | 14 |
| 55 | 171041 | M4a-BZ-CG-TW15 (2) | Pit C | 0.9527 | 0.0028 | 380 | 30 | 1877 | 1939 | 1939 | 97.4 | 93 |
| 56 | 171039 | M6d-BZ-CG-TW15 | Pit C | 0.9496 | 0.0028 | 400 | 30 | 1875 | 1927 | 1927 | 85.6 | 6 |
| 57 | 171038 | M8a-BZ-CG-TW15 | Pit C | 0.9555 | 0.0033 | 355 | 35 | 1877 | 1943 | 1943 | 79.8 | 7 |
| 58 | 171040 | M8e BZ -CG-TW15 (2) | Pit C | 0.9436 | 0.0031 | 455 | 35 | 1872 | 1910 | 1910 | 79.6 | 7 |
| 59 | 171091 | M8e-BZ-CG-TW15 | Pit C | 0.9523 | 0.0029 | 380 | 30 | 1877 | 1937 | 1937 | 79.3 | 9 |
| 60 | 171037 | M8f-BZ-CG-TW15 | Pit C | 0.9513 | 0.0028 | 390 | 30 | 1875 | 1934 | 1934 | 74.8 | 8 |
| 61 | 171048 | M8f-BZ-CG-TW15 (2) | Pit C | 0.9499 | 0.0028 | 400 | 30 | 1876 | 1928 | 1928 | 79.6 | 7 |
| 62 | 171044 | M9a-BZ-CG-TW15 | Pit C | 0.9460 | 0.0028 | 435 | 30 | 1872 | 1916 | 1916 | 85.6 | 6 |

Table S1: Data were reported as Fraction Modern Δ^14^C, and conventional radiocarbon age as defined in Stuiver and Polach [102]. Reported results include a sample preparation background based on contemporaneous measurements of samples of 14C-free calcite, and a mass dependent fractionation correction using sample specific δ^13^C or an assumed δ^13^C value of -3±1. Data were calibrated to calendar years using OxCal Version 4.2 against the Marine 13 calibration curve [104-105]. A code for OxCal v4.2 that accounts for the post-bomb local/regional offset in radiogenic carbon at Coral Gardens [88] was created using data from nearby Glovers Reef (16° 50' N, 87° 50' W). Within OxCal an agreement index is calculated which is a measure of the agreement between the model prior and the probability or likelihood of the observed data. In essence this index, A_comb_, is similar to a Χ^2^ test.
