# Supplementary material for: Coral Gardens Reef, Belize: A refugium in the face of Caribbean-wide Acropora spp. coral decline
Source: PLoS One. 2020 Sep 30;15(9):e0239267. doi: 10.1371/journal.pone.0239267 (PMC7526931; doi:10.1371/journal.pone.0239267)
Supplement: S2 Table — (DOCX) [file pone.0239267.s002.docx]

Table S2. MC ICP-MS ^230^Th age data for 35 dead coral samples collected from Coral Gardens, Belize.

| Sample name | Sample location | Sample wt.(g) | U (ppm) | ^232^Th (ppb) | (^230^Th/^232^Th) | (^230^Th/ ^238^U) | Corr. δ^238^U^1^ | Uncorr. ^230^Th Age (ka) | Date of chemistry | Corr. year (AD)^2^ |
| --- | --- | --- | --- | --- | --- | --- | --- | --- | --- | --- |
| F7a-BZ-CG-TW15 | Living | 0.17158 | 3.2160 ± 0.0020 | 0.03604 ± 0.00017 | 14.69 ± 0.34 | 0.0000543 ± 0.0000012 | 143.9 ± 1.2 | 0.00517 ± 0.00012 | 2015.53 | 2015.0 ± 0.9 |
| T5Sb-BZ-CG-TW15 | Modern Canopy | 0.18573 | 3.2080 ± 0.0028 | 0.05258 ± 0.00029 | 52.09 ± 0.91 | 0.0002814 ± 0.0000047 | 148.6 ± 1.1 | 0.02671 ± 0.00044 | 2015.58 | 1993.6 ± 1.0 |
| T5Sd-BZ-CG-TW15 | Modern Canopy | 0.27624 | 3.2696 ± 0.0019 | 0.06616 ± 0.00042 | 53.54 ± 1.02 | 0.0003571 ± 0.0000064 | 146.4 ± 1.2 | 0.03397 ± 0.00061 | 2015.58 | 1986.3 ± 1.1 |
| A2a-BZ-CG-TW15 | Pit C | 0.20141 | 3.4614 ± 0.0015 | 0.13827 ± 0.00025 | 18.91 ± 0.49 | 0.0002490 ± 0.0000064 | 148.6 ± 0.9 | 0.02365 ± 0.00061 | 2015.58 | 1996.8 ± 1.2 |
| A6a-BZ-CG-TW15 | Pit C | 0.16644 | 3.4903 ± 0.0015 | 0.14287 ± 0.00028 | 26.51 ± 0.40 | 0.0003576 ± 0.0000053 | 146.3 ± 1.2 | 0.03402 ± 0.00051 | 2015.58 | 1986.4 ± 1.1 |
| G3a-BZ-CG-TW15 | Pit C | 0.18661 | 3.3499 ± 0.0031 | 0.21509 ± 0.00027 | 35.25 ± 0.38 | 0.0007459 ± 0.0000080 | 149.4 ± 0.8 | 0.07079 ± 0.00076 | 2015.58 | 1950.2 ± 1.3 |
| M4a-BZ-CG-TW15 | Pit C | 0.15431 | 3.0876 ± 0.0015 | 0.06437 ± 0.00015 | 159.59 ± 1.53 | 0.001097 ± 0.000010 | 148.3 ± 0.9 | 0.10418 ± 0.00098 | 2015.58 | 1916.4 ± 1.4 |
| M8f-BZ-CG-TW15 | Pit C | 0.16442 | 3.3210 ± 0.0015 | 0.28527 ± 0.00038 | 37.24 ± 0.37 | 0.001054 ± 0.000010 | 148.3 ± 0.9 | 0.10017 ± 0.00099 | 2015.58 | 1921.3 ± 1.5 |
|  |  |  |  |  |  |  |  |  |  |  |
| F10c_BZ-CG-TW15 | Modern Canopy | 0.15188 | 3.2473 ± 0.0025 | 0.01524 ± 0.00014 | 60.30 ± 1.72 | 0.0000933 ± 0.0000025 | 150.5 ± 0.8 | 0.00884 ± 0.00024 | 2015.84 | 2011.4 ± 0.9 |
| F10a_BZ-CG-TW15 | Modern Canopy | 0.1522 | 3.1943 ± 0.0015 | 0.01717 ± 0.00024 | 60.25 ± 1.57 | 0.0001067 ± 0.0000024 | 148.1 ± 1.3 | 0.01014 ± 0.00023 | 2015.84 | 2010.2 ± 0.9 |
| T5Nc_BZ-CG-TW15 | Modern Canopy | 0.15589 | 3.0778 ± 0.0019 | 0.06231 ± 0.00016 | 17.46 ± 0.38 | 0.0001165 ± 0.0000025 | 148.1 ± 1.2 | 0.01106 ± 0.00024 | 2015.84 | 2009.7 ± 1.0 |
| F8a_BZ-CG-TW15 | Modern Canopy | 0.15505 | 3.2975 ± 0.0018 | 0.02876 ± 0.00020 | 40.39 ± 1.07 | 0.0001161 ± 0.0000030 | 147.3 ± 1.3 | 0.01104 ± 0.00028 | 2015.84 | 2009.2 ± 0.9 |
| F3a_BZ-CG-TW15 | Modern Canopy | 0.15345 | 3.4656 ± 0.0021 | 0.12088 ± 0.00022 | 10.64 ± 0.28 | 0.0001223 ± 0.0000032 | 146.9 ± 1.1 | 0.01163 ± 0.00030 | 2015.84 | 2008.9 ± 1.0 |
| T5Ed_BZ-CG-TW15 | Modern Canopy | 0.15781 | 3.1612 ± 0.0022 | 0.04637 ± 0.00017 | 26.31 ± 0.83 | 0.0001272 ± 0.0000040 | 146.6 ± 1.1 | 0.01210 ± 0.00038 | 2015.84 | 2008.5 ± 1.0 |
| T5Ee_BZ-CG-TW15 | Modern Canopy | 0.15237 | 3.1812 ± 0.0019 | 0.06522 ± 0.00015 | 19.14 ± 0.49 | 0.0001294 ± 0.0000033 | 147.8 ± 1.1 | 0.01229 ± 0.00031 | 2015.84 | 2008.4 ± 1.0 |
| T5Ef_BZ-CG-TW15 | Modern Canopy | 0.15232 | 3.0888 ± 0.0022 | 0.08873 ± 0.00026 | 14.03 ± 0.31 | 0.0001328 ± 0.0000029 | 149.2 ± 1.1 | 0.01260 ± 0.00028 | 2015.84 | 2008.3 ± 1.1 |
| T5Eb_BZ-CG-TW15 | Modern Canopy | 0.15289 | 3.1770 ± 0.0020 | 0.07202 ± 0.00018 | 21.20 ± 0.59 | 0.0001584 ± 0.0000044 | 149.8 ± 1.2 | 0.01502 ± 0.00042 | 2015.84 | 2005.7 ± 1.1 |
| F9a_BZ-CG-TW15 | Modern Canopy | 0.16957 | 3.2427 ± 0.0017 | 0.03285 ± 0.00021 | 48.59 ± 1.15 | 0.0001622 ± 0.0000037 | 148.2 ± 0.9 | 0.01541 ± 0.00035 | 2015.84 | 2005.0 ± 1.0 |
| T5Wa_BZ-CG-TW15 | Modern Canopy | 0.15424 | 3.1664 ± 0.0015 | 0.14451 ± 0.00042 | 12.50 ± 0.25 | 0.0001880 ± 0.0000038 | 150.2 ± 0.9 | 0.01783 ± 0.00036 | 2015.84 | 2003.3 ± 1.1 |
| T5Na_BZ-CG-TW15 | Modern Canopy | 0.16181 | 3.0933 ± 0.0020 | 0.19431 ± 0.00036 | 10.19 ± 0.23 | 0.0002109 ± 0.0000047 | 147.5 ± 1.3 | 0.02005 ± 0.00045 | 2015.84 | 2001.5 ± 1.2 |
| F1a_BZ-CG-TW15 | Modern Canopy | 0.15291 | 3.1376 ± 0.0018 | 0.11831 ± 0.00028 | 21.01 ± 0.56 | 0.0002611 ± 0.0000070 | 146.3 ± 1.3 | 0.02484 ± 0.00067 | 2015.84 | 1996.2 ± 1.3 |
| T5We_BZ-CG-TW15 | Modern Canopy | 0.16096 | 3.2549 ± 0.0019 | 0.09496 ± 0.00026 | 28.80 ± 0.61 | 0.0002769 ± 0.0000058 | 150.0 ± 0.8 | 0.02626 ± 0.00055 | 2015.84 | 1994.5 ± 1.1 |
| T5Wc_BZ-CG-TW15 | Modern Canopy | 0.15198 | 3.2097 ± 0.0019 | 0.08761 ± 0.00030 | 30.89 ± 0.53 | 0.0002779 ± 0.0000047 | 148.4 ± 1.0 | 0.02639 ± 0.00044 | 2015.84 | 1994.4 ± 1.1 |
| F1b_BZ-CG-TW15 | Modern Canopy | 0.15895 | 3.1918 ± 0.0015 | 0.14726 ± 0.00032 | 18.98 ± 0.36 | 0.0002887 ± 0.0000055 | 147.5 ± 1.0 | 0.02744 ± 0.00052 | 2015.84 | 1993.7 ± 1.2 |
| A1b_BZ-CG-TW15 | Pit C | 0.15179 | 3.3969 ± 0.0030 | 0.14366 ± 0.00029 | 20.87 ± 0.48 | 0.0002909 ± 0.0000066 | 146.3 ± 1.4 | 0.02768 ± 0.00063 | 2015.84 | 1993.1 ± 1.2 |
| T5Wb_BZ-CG-TW15 | Modern Canopy | 0.17223 | 3.2901 ± 0.0015 | 0.10452 ± 0.00036 | 28.52 ± 0.53 | 0.0002986 ± 0.0000054 | 147.9 ± 1.1 | 0.02837 ± 0.00052 | 2015.84 | 1992.4 ± 1.1 |
| T5Sf_BZ-CG-TW15 | Modern Canopy | 0.15047 | 3.2681 ± 0.0012 | 0.26047 ± 0.00055 | 12.41 ± 0.22 | 0.0003259 ± 0.0000057 | 147.1 ± 0.9 | 0.03098 ± 0.00054 | 2015.84 | 1990.7 ± 1.3 |
| T5Wd_BZ-CG-TW15 | Modern Canopy | 0.16769 | 3.2350 ± 0.0028 | 0.13326 ± 0.00030 | 25.06 ± 0.40 | 0.0003402 ± 0.0000054 | 148.1 ± 1.1 | 0.03232 ± 0.00051 | 2015.84 | 1988.7 ± 1.2 |
| T5Sd_BZ-CG-TW15 | Modern Canopy | 0.15314 | 3.2571 ± 0.0036 | 0.06729 ± 0.00028 | 55.82 ± 1.09 | 0.0003801 ± 0.0000073 | 150.9 ± 1.3 | 0.03602 ± 0.00069 | 2015.84 | 1984.5 ± 1.2 |
| T5Si_BZ-CG-TW15 | Modern Canopy | 0.1535 | 3.2507 ± 0.0030 | 0.26151 ± 0.00051 | 15.49 ± 0.22 | 0.0004107 ± 0.0000058 | 149.7 ± 1.0 | 0.03896 ± 0.00055 | 2015.84 | 1982.7 ± 1.3 |
| D4a_BZ-CG-TW15 | Pit C | 0.16107 | 3.4218 ± 0.0021 | 0.25277 ± 0.00052 | 23.40 ± 0.23 | 0.0005698 ± 0.0000055 | 150.1 ± 1.2 | 0.05404 ± 0.00053 | 2015.84 | 1967.3 ± 1.2 |
| A2b_BZ-CG-TW15 | Pit C | 0.15159 | 3.5535 ± 0.0026 | 3.1496 ± 0.0036 | 2.736 ± 0.037 | 0.000799 ± 0.000011 | 149.0 ± 1.0 | 0.0759 ± 0.0010 | 2015.84 | 1960.8 ± 4.3 |
| M8a_BZ-CG-TW15 | Pit C | 0.15458 | 3.2504 ± 0.0023 | 0.09039 ± 0.00032 | 78.90 ± 0.94 | 0.0007231 ± 0.0000083 | 152.1 ± 0.9 | 0.06847 ± 0.00079 | 2015.84 | 1952.2 ± 1.2 |
| F6a_BZ-CG-TW15 | Modern Canopy | 0.16154 | 3.3507 ± 0.0016 | *1.3783 ± 0.0019 | 7.475 ± 0.068 | 0.0010134 ± 0.0000092 | 148.6 ± 1.3 | 0.09625 ± 0.00088 | 2015.84 | 1931.6 ± 2.6 |
| J2a_BZ-CG-TW15 | Pit C | 0.15637 | 3.3545 ± 0.0025 | 0.29913 ± 0.00057 | 38.16 ± 0.39 | 0.001121 ± 0.000011 | 149.4 ± 0.9 | 0.1064 ± 0.0011 | 2015.84 | 1915.3 ± 1.6 |

Ratios in parentheses are activity ratios calculated from atomic ratios using ISOPLOT 3.75 (Ludwig 2012) using decay constants λ_238_= 1.55125 × 10^-10^ yr^-1^ (Jaffey et al. 1971), λ_234_ = (2.8262±0.0057) × 10^-6^ yr^-1^, λ_230_ = (9.158±0.028) × 10^-6^ yr^-1^ (Cheng et al. 2000). All values have been calculated after mean laboratory blank extraction. All errors reported in this table are quoted as 2σ.

^1.^δ^234^U(T)=δ^234^U(O)e ^λ234T^ where δ^234^U=[(^234^U/^238^U)-1] × 1000

^2.^To account for both hydrogenous and terrestrially derived ^230^Th_0_, the corrected (corr.) ^230^Th age of each sample was calculated using a sample specific non-radiogenic (^230^Th/^232^Th) value using the following equation in Clark et al. (2014):

$$\left(\frac{^{230}Th}{^{232}Th}\right)_{mix}=\left(\left(\frac{^{232}Th_{live}}{^{232}Th_{dead}}\right)\times\left(\frac{^{230}Th}{^{232}Th}\right)_{live}\right)+\left(\left(\frac{^{232}Th_{dead}-^{232}Th_{live}}{^{232}Th_{dead}}\right)\times\left(\frac{^{230}Th}{^{232}Th}\right)_{sed}\right)$$

where ^232^Th_dead_ is the measured ^232^Th value (ppb) in the individual dead coral sample. Hydrogenous and detrital values used in the equation reflect values obtained from *Porites* corals collected from the Palm Islands region, Great Barrier Reef (Clark et al. 2014), due to the unavailability of locally derived Belize coral sample values. ^232^Th_live_ is the mean ^232^Th value (ppb) measured in live *Porites* spp. coral samples collected from the Palm Islands, determined to be 0.95 ppb (N=12). ^230^Th/^232^Th_live_ represents or approximates the isotopic composition of the hydrogenous component in the live *Porites* spp. coral skeleton during growth with an activity value of 1.08 ± 20% (atomic ratio 5.85×10^-6^ ± 20%) ^230^Th/^232^Th_sed_ represents the detrital component incorporated into the coral skeleton post-mortem with a mean activity value of 0.61 ± 20% (atomic ratio 3.53 ×10^-6^ ± 20%) based on y-intercept values of ^230^Th/^232^Th versus ^238^U/^232^Th isochrons obtained from dead *Porites* collected from the Palm Islands.
